# Potential Early Identification of a Large Campylobacter Outbreak Using Alternative Surveillance Data Sources: Autoregressive Modelling and Spatiotemporal Clustering

**DOI:** 10.2196/18281

**Published:** 2020-09-17

**Authors:** Mehnaz Adnan, Xiaoying Gao, Xiaohan Bai, Elizabeth Newbern, Jill Sherwood, Nicholas Jones, Michael Baker, Tim Wood, Wei Gao

**Affiliations:** 1 Institute of Environmental Science and Research Porirua New Zealand; 2 Victoria University of Wellington Wellington New Zealand; 3 Hawke's Bay District Health Board Hawke's Bay New Zealand; 4 University of Otago Wellington New Zealand

**Keywords:** Campylobacter, disease outbreaks, forecasting, spatio-temporal analysis

## Abstract

**Background:**

Over one-third of the population of Havelock North, New Zealand, approximately 5500 people, were estimated to have been affected by campylobacteriosis in a large waterborne outbreak. Cases reported through the notifiable disease surveillance system (notified case reports) are inevitably delayed by several days, resulting in slowed outbreak recognition and delayed control measures. Early outbreak detection and magnitude prediction are critical to outbreak control. It is therefore important to consider alternative surveillance data sources and evaluate their potential for recognizing outbreaks at the earliest possible time.

**Objective:**

The first objective of this study is to compare and validate the selection of alternative data sources (general practice consultations, consumer helpline, Google Trends, Twitter microblogs, and school absenteeism) for their temporal predictive strength for Campylobacter cases during the Havelock North outbreak. The second objective is to examine spatiotemporal clustering of data from alternative sources to assess the size and geographic extent of the outbreak and to support efforts to attribute its source.

**Methods:**

We combined measures derived from alternative data sources during the 2016 Havelock North campylobacteriosis outbreak with notified case report counts to predict suspected daily Campylobacter case counts up to 5 days before cases reported in the disease surveillance system. Spatiotemporal clustering of the data was analyzed using Local Moran’s I statistics to investigate the extent of the outbreak in both space and time within the affected area.

**Results:**

Models that combined consumer helpline data with autoregressive notified case counts had the best out-of-sample predictive accuracy for 1 and 2 days ahead of notified case reports. Models using Google Trends and Twitter typically performed the best 3 and 4 days before case notifications. Spatiotemporal clusters showed spikes in school absenteeism and consumer helpline inquiries that preceded the notified cases in the city primarily affected by the outbreak.

**Conclusions:**

Alternative data sources can provide earlier indications of a large gastroenteritis outbreak compared with conventional case notifications. Spatiotemporal analysis can assist in refining the geographical focus of an outbreak and can potentially support public health source attribution efforts. Further work is required to assess the location of such surveillance data sources and methods in routine public health practice.

## Introduction

### Background

In August 2016, Havelock North, one of the 5 cities in the Hawke’s Bay region, New Zealand, was the site of a large waterborne outbreak of Campylobacter infection. This outbreak began on August 8, but a large number of cases were not known to the national notifiable disease surveillance system until August 14. By that time, more than a third of Havelock North residents had been infected with Campylobacter. This event led to serious interruption of daily life in the area and large economic costs [1,2].

The surveillance for notifiable diseases in New Zealand is predominantly passive, with laboratories and physicians notifying their local public health service through submission to the national notifiable disease surveillance system, EpiSurv [3]. There are inevitable delays from when people are exposed to an outbreak source (in this outbreak, the source was contaminated drinking water) to when they become ill, seek medical care, are diagnosed, and then notified to health authorities. There are usually further delays before an outbreak is recognized, investigated, and controlled. Therefore, notifiable disease reports are after the fact, and the information is typically delayed due to systematic information flow through traditional channels, for example, from physicians and laboratories.

Interest in considering alternative data sources for early prediction of such outbreaks was motivated by previously published work reporting on the use of data from internet search engines [4-7], crowd-sourced participatory disease surveillance systems [8,9], Twitter microblogs [5,10,11], news stories [12], school absenteeism reports [13,14], general practice (GP) consultations [15], consumer helpline calls [16,17], bank transactions [18], and numerous other sources. Location-aware applications have also been exploited for public and environmental health surveillance and crisis management [19,20] or to provide situational awareness and forecasting for disease outbreaks at the local level [20].

### Objectives

This study revisits the Havelock North Campylobacter outbreak to examine signals present in data sources that were not available to the public health team during the response. By analyzing temporal and spatiotemporal patterns in these alternative data sources, the study assesses the relative effectiveness and sensitivity of different data sources in detecting the outbreak earlier. First, we aim to assess the temporal predictive strength of modeled combinations of measures from the following daily alternative data sources: GP consultations, consumer health helpline calls, Google Trends, Twitter microblogs, and school absenteeism records. These models will be measured by the time gained (up to 5 days ahead) compared with the cases notified in the existing disease surveillance system, using multiple evaluation metrics. Second, we will examine city-level spatiotemporal patterns in measures from alternative data sources relative to notified case counts to identify clusters and outliers in both space and time over the outbreak period.

## Methods

### Ethics

The study protocol was approved by the Health and Disability Ethics Committee, New Zealand, under the protocol number NZ/1/6350114. The Twitter data used in this study were obtained under the Twitter terms and conditions and in agreement with its public privacy settings.

### Data Collection and Management

For the greater area affected by the outbreak (Hawkes Bay), we collected daily data for the entire 2016 calendar year from the data sources described in Table 1.

**Table 1:** Description of data sources used in analysis.

Source	Fields of interest	Data level used in analysis	Counts	References
Notified case count (New Zealand surveillance database EpiSurv)	Date of onset, testing, and notification for confirmed and probable cases of campylobacteriosis	Aggregated by notification date and city of residence in Hawkes Bay	1345	Ministry of Health New Zealand [3]
General practice consultations (HealthStat)	Visits for gastrointestinal complaints	Individual with visit date, age, and sex, for entire Hawkes Bay District Health Board area only	772	Cumming J and Gribben B [21]
Consumer helpline (HealthLine) calls	Consumer calls concerning gastrointestinal complaints	Individual with call date, age, sex, and residential city in Hawkes Bay	1196	St George IM and Cullen MJ [22]
Google Trends	User queries with keywords for gastrointestinal complaints	Normalized counts aggregated by date, query keyword, and Google Trends normalized count for entire Hawkes Bay District Health Board area only	Not applicable	Google Trends [23]
Twitter microblogs (from Gnip Historical PowerTrack service)	Tweets with keywords for gastrointestinal complaints	Individual tweets geocoded to cities in Hawkes Bay	191	Gnip [24]
School absenteeism records (from individual schools)	Absence owing to illness or any valid reason	Aggregated by schools for the 5 schools providing data, areas represented: Havelock North, Napier, and Hastings	23,836	Ministry of Education, New Zealand [25]

#### Notified Case Count

We extracted confirmed and suspected cases of campylobacteriosis in Hawkes Bay from EpiSurv [3] and aggregated them by report date and city-level locations. EpiSurv is the core surveillance system used for monitoring the occurrence of notifiable infectious diseases such as campylobacteriosis and detecting increases that may indicate an outbreak in New Zealand [26]. We refer to these data as *notified case counts* and use them as the main comparator for assessing the potential value of alternative surveillance data sources.

#### GP Consultations

Daily data on consultations with GPs were collected through HealthStat. This system automatically monitors the number of people who consult primary care medical practitioners based on automated extracts of GP-coded data from computerized practice management systems [21]. The data we used were the daily counts of those who consulted for gastroenteritis.

#### Consumer Helpline Calls

Consumer helpline data were collected from HealthLine, which is a free national 24-hour 0800 telephone health advice service funded by the New Zealand Ministry of Health [22]. Calls made to HealthLine are triaged using electronic clinical decision support software. The data collected are a daily count and the city-level location of all phone calls made to HealthLine by people reporting symptoms of gastrointestinal illness. A list of the symptoms used is included in Multimedia Appendix 1.

#### Google Trends

Google Trends provides a time series index of the volume of queries users enter into Google in a given geographic area [23]. We collected daily Google Trends data for a range of keywords that could be used to search for information regarding any gastrointestinal illness (see Multimedia Appendix 2 for a list of keywords). These Google Trends data were downloaded within a single day, as Google varies the signal display over time. Google Trends data for the selected keywords were assessed for correlation and cross correlation with the notified case counts for up to 10 previous days, and those keywords with correlations over 0.03 were chosen for the further analysis: “campylobacter,” “diarrhoea,” “diarrhea,” “gastro,” “gastroenteritis,” “puke,” and “vomiting.” Pearson correlation and cross correlation (same day and lagged) of these keywords in Google Trends with notified case counts of campylobacteriosis (January 2016 to July 2016) are presented in Multimedia Appendix 3.

#### Twitter Microblogs

Twitter is a free social networking and microblogging service that enables millions of users to send and read each other's tweets, or short, 140-character messages. Registered users collectively send more than 200 million tweets a day. Twitter accounts are by default public and visible to all (even to unregistered visitors using the Twitter website). Users can restrict their account settings to private, in which case their contents can only be visible to approved followers.

In a previous study, we obtained Twitter data from Gnip, their licensed data provider, through their Historical PowerTrack service [24]. In contrast to the publicly available Twitter data stream (Twitter application programing interface), which provides approximately 1% of all real-time tweets, the Historical PowerTrack provides search access to 100% of all publicly available tweets as well as metadata associated with each tweet. Tweets generated between April 2012 and March 2017 were collected from PowerTrack. They contained one or more gastrointestinal-related keywords and were assigned a country code of New Zealand in the Tweet or in the user profile location. The Gnip Query to collect Twitter data is included in Multimedia Appendix 4. A total of 131,843 records were obtained. These data were first geocoded using the latitude and longitude of the tweet. If the tweet location was missing, the profile latitude and longitude were used.

Twitter feeds were classified by developing a supervised machine learning classifier using the Naïve Bayes algorithm in Python. A total of 10,000 random tweets were manually labeled as (1) gastrointestinal illness, (2) other infectious illness, and (3) irrelevant tweets. A tweet was labeled “gastrointestinal illness” when its content described a recent account of infectious gastrointestinal illness, “infectious illness” for tweets that described a recent account of other infectious illnesses, and “irrelevant” for tweets that did not fit in the other 2 categories. This training set was used to train the machine learning classifier, which was then used to classify the complete Twitter data. This classifier was evaluated on 1000 randomly selected and manually labeled tweets that were not included in the training set. Precision, recall, and F1 scores were calculated to evaluate the performance of the classifier. Precision is the ratio of observations judged relevant to the total observations predicted as relevant, recall is the ratio of observations judged relevant out of total relevant observations, and F1 is the weighted average of precision and recall [27]. The classification method obtained a precision of 0.813, recall of 0.803, and F1 score of 0.804. We applied this developed supervised classifier to the data from the Hawkes Bay region for the period of January 1, 2016, to December 31, 2016.

#### School Absenteeism

We collected school absenteeism data from 5 schools in Hawke’s Bay: 2 from Havelock North, 2 from Hastings, and 1 from Napier. These included 4 primary schools and 1 secondary school. Primary school data had a reason for absence code, so we included data for codes related to illness and/or any justified absence. Absenteeism codes are listed in Multimedia Appendix 5. For the secondary school, all absenteeism counts were included without any subcoding. Havelock North and Hastings were the areas primarily affected by the outbreak, whereas the Napier school served as a control.

A daily time series with cumulative counts from all the previously mentioned data sources was constructed. For the school data set, days covering the school holidays were removed from the analysis. In all data sources, missing data values were estimated by interpolation of observational data. These adjustments were made to reduce the impact of missing data in the analysis.

### Statistical Analysis

#### Correlation and Cross Correlation

To assess whether the selected data sources could have predicted this Campylobacter outbreak earlier, we used Pearson correlation statistics to calculate correlations between daily counts of these alternative surveillance measures and daily counts of notified cases. Correlations were calculated for the notified case count with the alternative measure on the same day as well as with up to a 10-day negative lag for each alternative measure (ie, correlating the notified case count on day t with the alternative measure on day t−10, t−9, etc; Table 2). Using this method, a significant correlation with the count on the same day indicates that the peak occurs at the same time [28], and the cross correlation at a specific lag of *x* days indicates that the peak in the alternative measure occurs *x* days before the peak in notified cases.

**Table 2:** Correlation and lagged transformed correlation of alternative predictors with notified case counts of campylobacteriosis.

Data source	Number of days that alternative measures are lagged before notifiable counts										
	0 days	−1 day	−2 days	−3 days	−4 days	−5 days	−6 days	−7 days	−8 days	−9 days	−10 days
GP^a^ consultations	0.5^b^	0.43^b^	0.39^b^	0.26^b^	0.17^b^	0.14^b^	0.09	0.05	0.04	0.01	0.01
Consumer helpline	0.44^b^	0.59^b^	0.67^b^	0.64^b^	0.55^b^	0.37^b^	0.2^b^	0.12^b^	0.1	0.07	0.07
Google Trends	0.13^b^	0.16^b^	0.22^b^	0.22^b^	0.21^b^	0.17^b^	0.21^b^	0.21^b^	0.16^b^	0.08	0.02
Twitter microblogs	0.11^b^	0.21^b^	0.31^b^	0.25^b^	0.21^b^	0.07	0	−0.01	0	−0.03	0
School absenteeism	0.3^b^	0.48^b^	0.64^b^	0.7^b^	0.52^b^	0.35^b^	0.21^b^	0.2^b^	0.17^b^	0.18^b^	0.15^b^

^a^GP: general practice.

^b^Statistically significant correlation coefficient >0.1.

#### Models

To forecast daily suspected cases of campylobacteriosis, a collection of multivariable autoregressive integrated moving average (ARIMA) models were constructed. These models were found to be a good tool for the prediction of communicable disease incidences [5,6,29-32]. These models are denoted as ARIMA(p,d,q), where parameters p, d, and q are non-negative integers; p is the number of autoregressive terms, d is the degree of differencing needed for stationarity, and q is the moving average component of the model. Data from January 1 to July 31, 2016, were used for model development. Model identification for ARIMA was initiated using the R statistical function auto.arima, which uses the Bayes information criterion to determine the orders p and q and the Phillips-Perron unit root test for determining the order d*.*

These models used the negative lagged (day −1 to day −10) daily counts for each alternative measure (Table 2) and the nonlagged notified case counts as covariates. We computed various permutations using different combinations of covariates and chose the optimal combination of covariates using the root mean square error (RMSE). The autocorrelation and partial autocorrelation plots of the models obtained from auto.arima were examined to further adjust the range of ARIMA (p and q) parameters. In addition to the models that used the aforementioned data streams as covariates, we built baseline models with only notified case counts for comparison and context. We considered models that only used historical observation of Campylobacter cases to predict cases on the subsequent days and models that incorporated information from the various alternative data streams to compare their predictive abilities during the volatile peak of the outbreak.

Models were thus evaluated for their predictive performance during the test period from July 31 to August 30, 2016. For each model, we report 3 evaluation metrics: the Pearson correlation (ρ), RMSE, and the relative root mean square error (rRMSE) of the predictions. ρ is a measure of the linear dependence between two variables during a period. RMSE is a measure of the difference between the predicted and true values. rRMSE is a measure of the percent difference between the predicted and true values. The equations for these measures are given below:

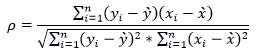

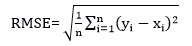

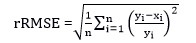







where y_i_ denotes the observed value of the notified Campylobacter cases at time t_i_, x_i_ denotes the predicted value by any model at time t_i_, 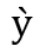 denotes the mean of the observed values, and 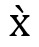 denotes the mean of the predicted values.

#### Spatiotemporal Clustering

Sources that included city-level locations (notified cases, school absenteeism, consumer helpline, and Twitter feeds) were used for spatiotemporal analysis. To understand the spatial and temporal trends of the event data, we broke them up into a series of time snapshots, using the space-time cube method [33]. We applied this method to the data for August 2016 from Havelock North and Hastings, the two largely affected cities in the outbreak.

We used a Local Outlier Analysis tool in ArcGIS (Esri) to identify locations that were statistically different from their neighbors in both space and time. This tool generates Anselin Local Moran’s I [34] statistics for each space-time window. These statistics have been used for spatial outlier detection in domains such as emergency management [35,36], epidemiology [37], and economics [38]. A Local Moran’s I with a negative value (representing high-low or low-high autocorrelation) suggests dissimilarity with neighbors; hence, an outlier, with a positive value (representing high-high or low-low autocorrelation) suggests similarity and a zero value suggests randomness. A *P* value less than .05 indicates that the cluster or outlier is statistically significant [39]. Twitter was found to be insufficient in terms of spatialized city-level data (with no tweet from Havelock North and only 4 from Hastings during the outbreak period) to generate Local Moran’s I statistics and hence was excluded from this analysis. The analysis was performed using ArcGIS Pro version 2.1.

## Results

### Relationship Between Notified Cases and Alternative Data

All alternative surveillance measures correlated significantly with notified Campylobacter cases on the same day. Many of these alternative surveillance measures also demonstrated strong correlations when lagged 1 to 8 days before notified cases. Indeed, the correlation ranged from 0.14 to 0.43 for up to 5 days of lag for GP consultations, 0.12 to 0.67 for up to 7 days of lag for consumer helpline inquiries, 0.16 to 0.22 for up to 8 days of lag for Google Trends, 0.21 to 0.31 for up to 4 days of lag for Twitter, and 0.15 to 0.7 for up to 10 days of lag for school absenteeism (Table 2).

### ARIMA Models

The final ARIMA models and the covariates of alternative data sources with their in-sample error measure of RMSE are summarized in Table 3. We found multiple models suitable for prediction: school absenteeism performed best (average RMSE: 1.00) with ARIMA (5,1,3) for forecasting 1 to 2 days ahead and ARIMA (5,0,2) for forecasting 3 to 5 days ahead, followed by Google Trends (average RMSE: 1.07) with ARIMA (2,0,0) for forecasting up to 5 days ahead. GP consultation was found to have an average RMSE of 1.04, with ARIMA (3,0,1) for forecasting for the following day and ARIMA (2,0,0) for forecasting 2-5 days ahead. Twitter had an average RMSE of 1.08 and HealthLine had an average RMSE of 1.084 when used as the covariates in the models for predicting notified case counts.

**Table 3:** Autoregressive integrated moving average models with time-lagged covariates used with alternative data sources for forecasting 1 to 5 days ahead.

Alternative data source and forecast step		Time-lagged covariates, days^a^		ARIMA^b^ order^c^		RMSE^d^	
**GP^e^ consultations**							
	1 day		1 to 10		3,0,1		1.01
	2 days		2 to 10		2,0,0		1.04
	3 days		3 to 10		2,0,0		1.04
	4 days		4 to 10		2,0,0		1.05
	5 days		5 to 10		2,0,0		1.06
**Consumer helpline**							
	1 day		1, 2, 3, 4, 5, 6, 7, 8, 10		3,0,2		1.08
	2 days		2, 3, 5, 6, 7, 8, 10		3,0,2		1.08
	3 days		3, 4, 5, 6, 7, 8, 10		3,0,2		1.08
	4 days		4, 6, 7, 8, 9, 10		3,0,2		1.09
	5 days		6, 7, 8, 9, 10		3,0,2		1.09
**Google Trends**							
	1 day		1 to 10		2,0,0		1.07
	2 days		2 to 10		2,0,0		1.08
	3 days		3 to 10		2,0,0		1.08
	4 days		4 to 10		2,0,0		1.08
	5 days		5 to 10		2,0,0		1.08
**Twitter**							
	1 day		1 to 10		4,0,1		1.07
	2 days		2 to 10		5,0,2		1.08
	3 days		3 to 10		3,0,2		1.08
	4 days		4 to 10		2,0,2		1.09
	5 days		5 to 10		2,0,2		1.09
**School absenteeism**							
	1 day		1 to 10		5,1,3		0.94
	2 days		2 to 10		5,1,3		0.94
	3 days		3 to 10		5,1,3		0.94
	4 days		4 to 10		5,0,2		1.09
	5 days		5 to 10		5,0,2		1.09

^a^Lagged covariates refer to the time-lagged independent variables of alternative data source.

^b^ARIMA: autoregressive integrated moving average.

^c^ARIMA order (p,d,q) refers to the number of autoregressive terms, degree of differencing, and moving average components of the model.

**^d^**RMSE**:** root mean square error.

^e^GP: general practice.

**Figure 1:**
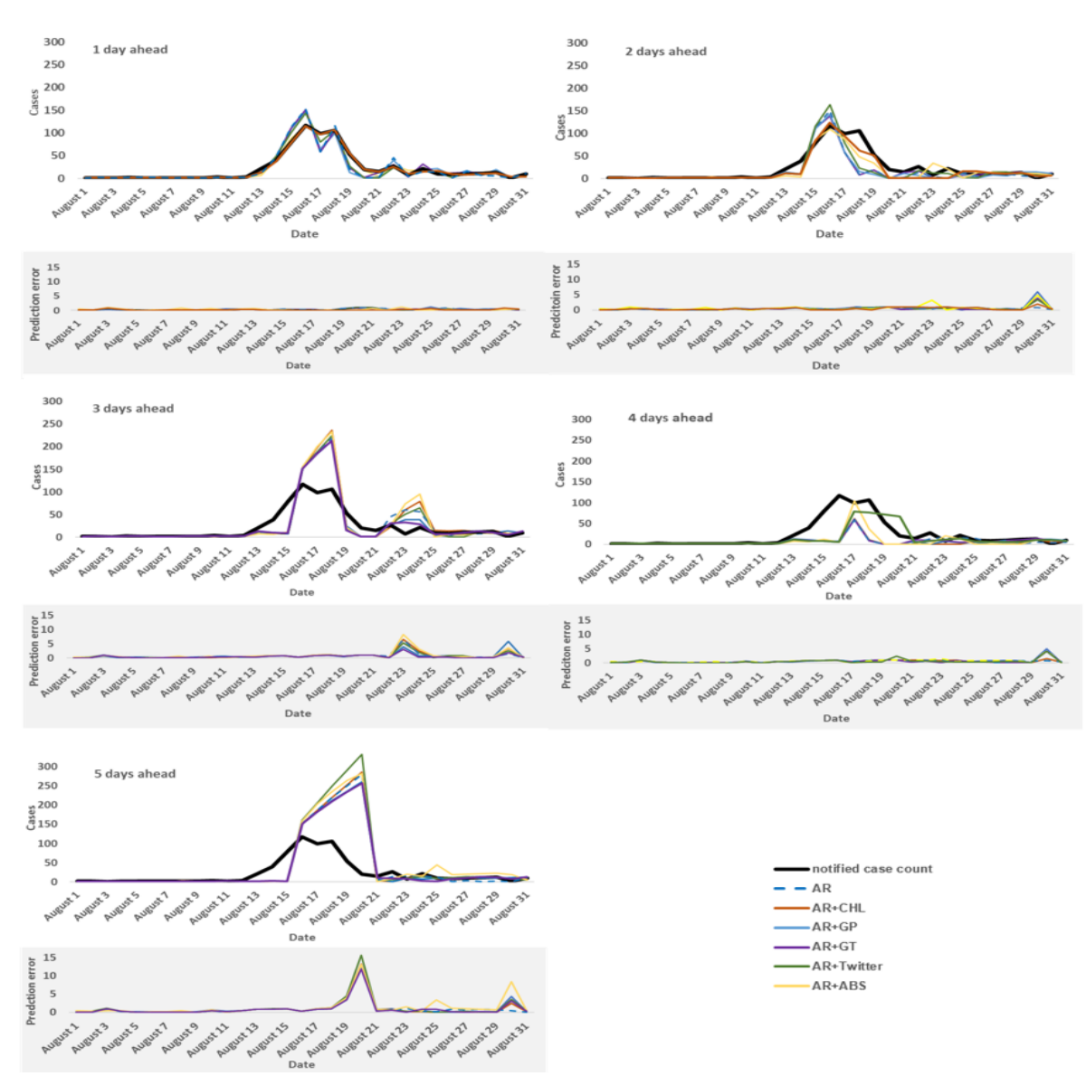
Actual notified case counts and prediction results 1 to 5 days ahead for all developed models, with their prediction errors based on relative root mean square error. The best model performance with the lowest prediction error (relative root mean square error) in each time series is shown as a bold line. ABS: abseentism; AR: autoregressive; CHL: consumer helpline; GP: general practice; GT: Google Trends.

We produced predictions for 1 to 5 days ahead during the outbreak (ie, the testing period) using the models in Table 3 and with the baseline models that used only autoregressive notified case counts. The daily estimations of the models with autoregressive (AR) information of notified case counts, AR with Google Trends (AR+GT), AR with consumer helpline (AR+CHL), AR with GP consultations (AR+GP), AR with school absenteeism (AR+ABS), and AR with Twitter (AR+Twitter) are presented in Figure 1.

Table 4 summarizes the predictive performance of the models during the test period for each of the 1-, 2-, 3-, 4-, and 5-day ahead predictions, as captured by the 3 evaluation metrics RMSE, rRMSE, and ρ. Although some model’s predictions showed good correlation with the notified case counts, their predictions showed large discrepancies from the true number of cases reported, as shown by the rRMSE. The rRMSE provides an estimate of the prediction error relative to the number of actual cases reported in each day over the evaluation period, and from our perspective, it provides a better measure of the quality of model prediction given the short time span of the outbreak.

**Table 4:** Root mean square error, relative root mean square error, and Pearson correlation for 1-, 2-, 3-, 4-, and 5-day ahead predictions during the test period (August 2016).

Model	1 Day			2 Days				3 Days				4 Days				5 Days		
	RMSE^a^	rRMSE^b^	ρ^c^	RMSE	rRMSE	ρ	RMSE		rRMSE	ρ	RMSE		rRMSE	ρ	RMSE		rRMSE	ρ
AR^d^	15.28	46.9	0.917	23.73	72.8	0.76	33.9		105.3	0.82	38.85		119.2	0.20	67.57		202	0.65
AR+CHL^e^	*2.74* ^f^ * *	*8.4* ^f^	*0.996* ^f^	*15.1* ^f^	*46.3* ^f^	*0.91* ^f^	39.74		123.5	0.79	38.14		117	0.28	68.51		204.8	0.64
AR+GP^g^	15.71	48.2	0.901	23.77	72.9	0.75	31.55		98	0.84	39.59		121.4	0.21	63.21		189	0.66
AR+GT^h^	12.9	39.6	0.933	22.5	69	0.76	*29.86* ^f^		*92.8* ^f^	*0.85* ^f^	37.84		116.1	0.21	*62.41* ^f^		*186.6* ^f^	*0.66* ^f^
AR+Twitter	11.61	35.6	0.951	22.67	69.5	0.80	35.63		110.7	0.81	*26.76* ^f^		*82.1* ^f^	*0.61* ^f^	80.83		241.7	0.62
AR+ABS^i^	4.74	14.5	0.989	15.97	49	0.89	38.68		120.2	0.81	47.26		145	0.28	71.5		213.8	0.65

^a^RMSE: root mean square error.

^b^rRMSE: relative root mean square error.

^c^ρ: Pearson correlation.

^d^AR: autoregressive.

^e^CHL: consumer helpline.

^f^Best performing model for a particular day on basis of the rRMSE.

^g^GP: general practice.

^h^GT: Google Trends.

^i^ABS: school absenteeism.

As seen in the evaluation metric values in Table 4, no model depending on a single data source performed best across all metrics or time periods. On the basis of the rRMSE, models that combined consumer helpline with autoregressive information (AR+CHL) outperformed all other models for 1 day and 2 days ahead predictions (rRMSE=8.4 and 46.3, respectively). Meanwhile, models that combined Twitter with autoregressive information from notified cases (AR+Twitter) performed best for 4-day ahead prediction (rRMSE=82.1), and models that combined Google Trends with autoregressive information (AR+GT) performed best for 3- and 5-day ahead predictions (rRMSE=92.8 and 186.6, respectively). In all time periods, the model using only the historical case counts underperformed all the other models.

**Figure 2:**
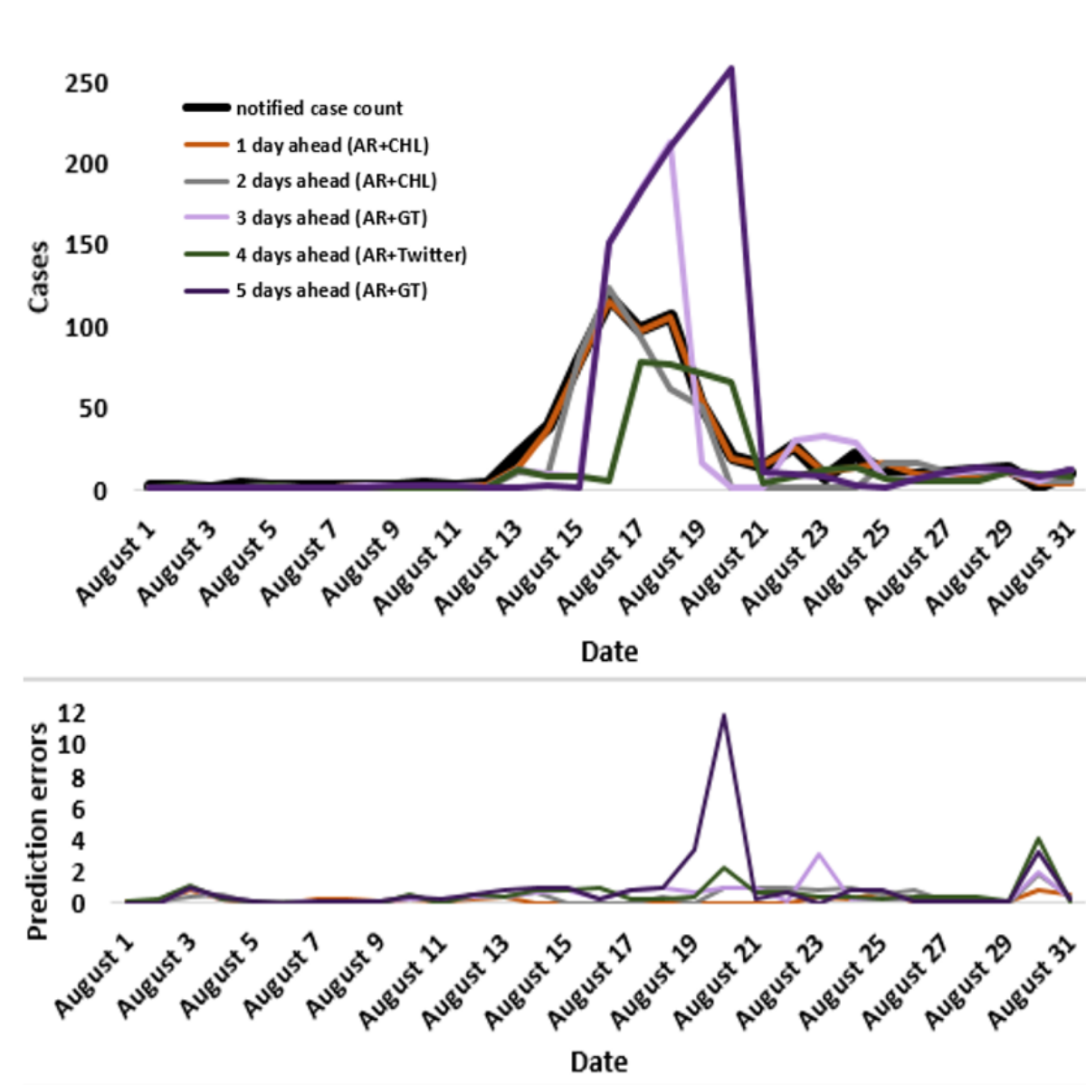
The daily estimations of the best performing models (lowest relative root mean square error) and their prediction errors during the testing period (August 2016). AR: autoregressive; CHL: consumer helpline; GT: Google Trends.

The out-of-sample (ie, using the data for the testing period) prediction with the best performing models for the 1, 2, 3, 4, and 5 days ahead time horizons and their prediction errors are shown in Figure 2. Across models, prediction accuracy decreased as predictions were made further days ahead, resulting in increases in rRMSE (and RMSE) and decrease in model correlations across time horizons. For example, for the best models, based on Google Trends, the prediction error nearly doubled from the 3-day to the 5-day forecast.

### Clustering and Cluster Detection

**Figure 3:**
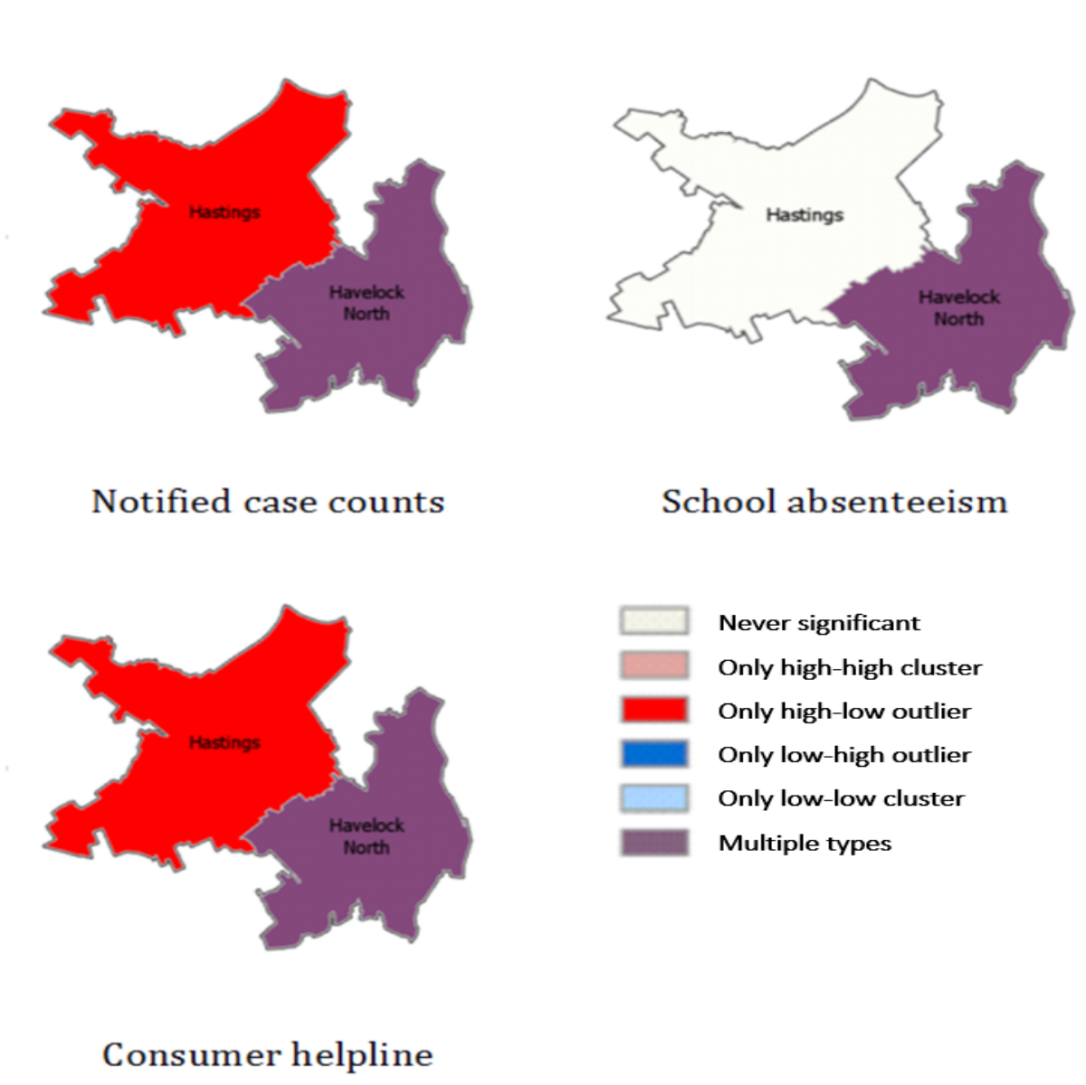
Cluster types in notified case counts, consumer helpline inquiries, and school’s absenteeism in Hastings and Havelock North. High-high cluster refers to high values surrounded by high values, high-low cluster refers to high values surrounded by low values, low-high cluster refers to low values surrounded by high values, and low-low cluster refers to low values surrounded by low values. Multiple Types refer to multiple cluster-type designations (ie, high high, low low, high low, and low high) through the time period.

The summarized cluster types in notified case counts, consumer helpline inquiries, and school absenteeism in Hastings and Havelock North are shown in Figure 3. Both notified case counts and consumer helpline inquiries indicated high-low outliers in Hastings and multiple cluster types (ie, high-high, low-low, high-low, and low-high) in Havelock North throughout the time period. The cluster types could not be identified in the Twitter data because of the limited availability of daily records in all 3 cities in the time period.

The prevalence of the designation Multiple Types did not illuminate trends or clusters in the data set. Therefore, we examined daily Local Moran’s I to compare the clustering between 2 cities during the outbreak (Table 5). Comparing the 2 cities, clustering in data sources was very weak in Hastings, compared with Havelock North. On the basis of Local Moran’s I, outliers were found in school absenteeism and consumer helpline (Moran’s I: −0.40 and −0.77, respectively) in Havelock North on August 11, 2016, which continued to grow in size until August 15, 2016. After 3 days, a stronger outlier appeared in the notified case counts (−2.17) from Havelock North. In Hastings, no significant cluster appeared in school absenteeism, a relatively weak cluster appeared in notified case counts, and a consumer helpline outlier appeared on August 14. These data suggest that the spatiotemporal indicators in consumer helpline and school absenteeism indicated the outbreak in Havelock North 3 days earlier than the notified surveillance data.

**Table 5:** Daily Local Moran’s I in school absenteeism, consumer helpline inquiries, and notified case counts in Havelock North and Hastings cities in August 2016.

Date	Havelock North			Hastings		
	School absenteeism	Consumer helpline	Notified case count	School absenteeism	Consumer helpline	Notified case count
	Moran’s I value, Z score	Moran’s I value, Z score	Moran’s I value, Z score	Moran’s I value, Z score	Moran’s I value, Z score	Moran’s I value, Z score
August 4, 2016	0.05 (−0.23)	0.08 (−0.29)	0.10 (−0.32)	0.03 (−0.16)	0.04 (−0.23)	0.08 (−0.29)
August 5, 2016	0.05 (−0.23)	0.08 (−0.29)	0.10 (−0.32)	0.04 (−0.23)	0.07 (−0.29)	0.09 (−0.32)
August 6, 2016	0.05 (−0.23)	0.08 (−0.29)	0.10 (−0.32)	0.05 (−0.23)	0.08 (−0.29)	0.10 (−0.32)
August 7, 2016	0.05 (−0.23)	0.08 (−0.29)	0.10 (−0.32)	0.05 (−0.23)	0.08 (−0.29)	0.10 (−0.32)
August 8, 2016	0.05 (−0.23)	0.08 (−0.29)	0.10 (−0.32)	0.05 (−0.23)	0.08 (−0.29)	0.10 (−0.32)
August 9, 2016	0.05 (−0.23)	0.08 (−0.29)	0.10 (−0.32)	0.05 (−0.23)	0.08 (−0.29)	0.10 (−0.32)
August 10, 2016	0.05 (−0.23)	0.08 (−0.29)	0.10 (−0.32)	0.04 (−0.19)	0.03 (−0.1)	0.09 (−0.29)
August 11, 2016	−*0.40 (1.74)*^a,^^b^	−*0.77 (2.71)*^a,^^b^	0 (0.01)	0.03 (−0.15)	0.01 (−0.1)	0.08 (−0.29)
August 12, 2016	−0.40 (−0.23)	-0.77 (−0.29)	0 (−0.32)	0.04 (−0.23)	0.03 (−0.29)	0.09 (−0.32)
August 13, 2016	0.05 (−0.23)	0.08 (−0.29)	0.10 (−0.32)	0.05 (−0.23)	0.08 (−0.29)	0.10 (−0.32)
August 14, 2016	−1.62 (7.08)^a^	−1.92 (6.71)^a^	−*2.17 (6.86)*^a,^^b^	*0.04 (* *−* *0.16)*	−*0.06 (0.22)*^b^	−*0.20 (0.64)*^b^
August 15, 2016	−1.62 (−0.23)	−1.92 (−0.29)	−2.17 (−0.32)	0.03 (−0.17)	-0.01 (−0.04)	0.56 (0.89)
August 16, 2016	0.05 (−0.23)	0.08 (−0.29)	0.10 (−0.32)	0.03 (−0.16)	0 (−0.04)	1.20 (1.37)
August 17, 2016	0.05 (0.23)	0.08 (−0.29)	0.10 (−0.32)	0.02 (−0.15)	0 (0.03)	1.20 (0.89)
August 18, 2016	0.05 (−0.23)	0.08 (−0.29)	0.10 (−0.32)	0.02 (−0.11)	0 (0.03)	0.31 (0.35)
August 19, 2016	0.05 (−0.23)	0.08 (−0.29)	0.10 (−0.32)	0.03 (−0.23)	−0.01 (−0.29)	−0.11 (−0.32)
August 20, 2016	0.05 (−0.23)	0.08 (−0.29)	0.10 (−0.32)	0.05 (−0.23)	0.08 (−0.29)	0.10 (−0.32)
August 21, 2016	0.05 (−0.23)	0.08 (−0.29)	0.10 (−0.32)	0.03 (−0.13)	0.01 (−0.04)	−0.08 (0.25)
August 22, 2016	0.05 (−0.23)	0.08 (−0.29)	0.10 (−0.32)	0.02 (−0.17)	0 (−0.04)	−0.05 (−0.19)
August 23, 2016	−0.10 (0.45)	−0.11 (0.37)	−0.11 (0.34)	0.03 (−0.18)	0 (−0.1)	−0.02(0.13)
August 24, 2016	0.21 (0.46)	0.14 (0.37)	0.12 (0.34)	0.03 (−0.16)	0.02 (−0.16)	−0.03 (−0.23)
August 25, 2016	0.14 (0.3)	0.14 (0.37)	0.23 (0.68)	0.03 (−0.16)	0.04 (−0.23)	0.06 (−0.29)
August 26, 2016	−0.07 (−0.23)	−0.11 (−0.29)	−0.22 (−0.32)	0.04 (−0.23)	0.07 (−0.29)	0.09 (−0.32)
August 27, 2016	0.05 (−0.23)	0.08 (−0.29)	0.10 (−0.32)	0.05 (−0.23)	0.08 (−0.29)	0.10 (−0.32)
August 28, 2016	−0.05 (0.2)	−0.01 (0.04)	−0.11 (0.34)	0.04 (−0.19)	0.03 (−0.1)	0.03 (−0.1)
August 29, 2016	−0.05 (−0.23)	−0.01 (−0.29)	−0.11 (−0.32)	0.04 (−0.23)	0.03 (−0.29)	0.03 (−0.32)
August 30, 2016	−0.02 (0.11)	−0.11 (0.37)	0.05 (−0.16)	0.05 (−0.23)	0.08 (−0.29)	0.10 (−0.32)

^a^Negative values of the Moran’s I value and corresponding Z scores greater than 1.96 indicate that there is a statistically significant spatial outlier.

^b^First day when the data source shows a spatial outlier.

## Discussion

### Principal Findings

The results show that alternative surveillance data sources can be used to predict an increase in notified Campylobacter cases up to 5 days before the outbreak would be detected via the notifiable disease surveillance system. Importantly, models that relied solely on available time-lagged notified case data were found to be no better than the models based on alternative data sources in predicting near–real-time Campylobacter cases. This finding further underscores the need for alternative real-time data sources such as consumer helpline and Google Trends.

Models that relied on consumer helpline calls provided 1 to 2 days of lead time before an increase in notified cases and consistently performed well, with low error rates. This finding suggests that consumer helpline data have potential utility for earlier detection of outbreaks of acute gastroenteritis. Qualitatively, this result is consistent with our expectations, as the consumer helpline and GP consultations are well-established services for those seeking medical attention in New Zealand [22] and can be expected to provide good predictors of potential cases.

The web data sources (Google Trends and Twitter) were found to be good estimators of Campylobacter cases, even earlier than consumer helpline data. For example, Google Trends reduced the prediction error by less than 6% compared with the next-best model (ie, with GP consultations) for 3-days ahead prediction, as shown in Table 4.

As seen in prediction studies for other diseases [7,31], the quality of predictions decreased as the time horizon of prediction increased. Specifically, for 1-day ahead predictions, we found that the model using consumer helpline combined with autoregressive terms (the AR+CHL model) performed best. The autoregressive terms generally help maintain predictions within a reasonable range, whereas the alternative data sources helped the models to respond more rapidly to sudden changes in the dynamics, a finding that has been documented in previous studies [7,40]. However, for 3- to 5- day ahead predictions, models that used data from Google Trends and Twitter performed best. Google search and Twitter activity appear to respond more rapidly to fluctuations in the dynamics of campylobacteriosis. Evidently, people affected by Campylobacter begin searching for gastrointestinal-related keywords when starting to have symptoms or when they may suspect a risk of exposure. This suggests that monitoring search activity may help track disease incidence.

Spatiotemporal analysis was also retrospectively able to confirm the area impacted by the outbreak. Havelock North and Hastings followed the same clustering in notified case counts and consumer helpline inquiries, whereas Hastings, which was not in the area most affected by the outbreak, had early peaks in consumer helpline inquiries and school absenteeism but fewer overall helpline calls and cases. Aggregating the time series data at the city level may immediately give indications of potential clusters, such as the one identified in Havelock North by Local Moran’s I statistics. In particular, primary clusters in school absenteeism and consumer helpline inquiries started on August 11, which was 3 days before the same type of cluster was found in notified case counts and a day earlier than actual public health response actions were initiated. Used prospectively, such spatiotemporal analysis could identify clusters and outbreaks earlier in their course than notification data [41].

### Limitations

There are limitations in our approach from inherent biases in the alternative data sources. Users of any of these services are not representative of the general population or those at risk of exposure to pathogens. Google search patterns and care seeking may reflect media coverage and situational awareness rather than the actual impact of the outbreak. Local media in regions with a large outbreak may react differently than the regions where these diseases are fewer in number. Thus, media attention has the potential to dramatically influence our daily predictions [42].

We used the correlation of keywords with notified cases to filter Google Trends data and to classify tweets, which improved the predictive values of these data sources. However, neither of these data sources can distinguish people who search or tweet because of awareness from those with infection. In addition, the static assessment of the predictive power of the included keywords can impose some limitations. Self-correcting keyword selection by dynamically reassessing the predictive power of each input variable, as discussed by McGough et al [7], could be used in the future to mitigate these limitations. The terms that peak due to high media attention could thus be excluded from the model if their relationship with case count information has weakened.

As mentioned in the Results section, there was insufficient Twitter data to use in the spatiotemporal analysis. However, tweets were only queried in English. With an already low tweet volume, capturing other languages such as Māori might be needed to refine models in the future. Furthermore, we relied on Twitter-generated coordinate information to capture local data. To overcome this limitation, future work could explore ways to geocode the data using location information in the tweet text [43]. For temporal analysis, only limited Twitter and school absenteeism data were available from the entire Hawke’s Bay region, presenting a clear limitation to the power of the analysis. It is encouraging that despite the limited school absenteeism data, it was still found to show statistically significant spatiotemporal clusters at the city level.

We are not advocating alternative data sources to replace traditional methods, but rather to complement them. For example, in the Havelock North outbreak, public health officials still required information that suggested an outbreak source (positive bacterial test from local water supply) to start control activities (boil water notice and chlorination of drinking water supply). Early signals from social media and HealthLine calls could have triggered efforts to investigate potential outbreak sources earlier. However, nontraditional surveillance carries with it the workload required to interpret and respond to signals, which can be extensive, as others have noted [44,45].

### Comparison With Previous Work

This study shows a number of improvements over previous methodologies using monthly or weekly data from alternative sources to predict disease incidence in the community [4-7,12,14,18], notably by using diverse daily data sources and combining with autoregressive modeling and spatiotemporal clustering to predict the incidence of gastrointestinal illness in a localized outbreak. Many researchers have used internet search queries to build prediction models in recent years. Bahk et al [6] used internet search query data for predicting weekly foodborne illness up to 2 months ahead of increases. Liu et al [4] used internet queries to predict weekly dengue fever outbreaks. Both of these analyses used Spearman r correlation to quantify the strength of associations between disease incidence and internet search queries. Similar to our study, Bahk et al [6] used the seasonal autoregressive integrative moving average (SARIMA) to develop their predictive models. However, Liu et al [4] used regression tree models to assess the threshold effects between the weekly disease incidence and internet search queries. Their results are consistent with those in this study, finding that internet search query data provided a timely data source for predicting the incidence of disease.

In addition to internet search volumes, some studies have used time-lagged data from Twitter to predict the incidence of diseases such as Zika [7] and influenza-like illness [5]. As in our study, McGough et al [7] used ARIMA and rRMSE to select the best model and found that Google typically performed better than Twitter for 2- and 3-week ahead predictions. However, rather than using static keywords, this study used a dynamic keyword selection method. Nagar et al [5] used an Englemen Granger co-integration test to make weekly predictions of influenza-like illness from time-lagged data sets containing Google, Twitter, and notified case counts. However, this study found that Twitter data produced better predictions than Google Trends data. Both of these studies found that time-lagged notified case data were not statistically significant in predicting cases in real time, in line with the results found in our study. In addition to regression models, Nagar et al [5] also used a spatial scan technique to identify areas with relatively higher risk of disease, comparable with the outlier analysis using Local Moran’s I, which we used to identify spatial outliers.

Dong et al [14] used diverse data sources including over-the-counter drug sales, search queries, and school absenteeism to estimate the correlation of these data sources with influenza activity. As in our study, they found that 1-week lagged data of internet search queries and school absenteeism showed the strongest correlation with laboratory-confirmed cases. However, they did not attempt to estimate the activity of disease in the community ahead of time. Widerström et al [17] used consumer helpline data and applied SARIMA to develop weekly predictive models for acute gastrointestinal illness and influenza-like illness. As in our study, consumer helpline data proved to be an important source for the early detection of outbreaks of these conditions. Wang et al [18] suggested the possibility of using bank transaction data with a simple moving average to monitor post outbreak disease spread, and they gave the Havelock North outbreak as an example; however, the use of such data for early warning of the outbreak was not very encouraging.

### Implications and Further Research

This study has further demonstrated that alternative surveillance data sources can identify large outbreaks of gastrointestinal illness a few days earlier than traditional surveillance methods. The lead time gained depends on the nontraditional surveillance data source used, with onset of symptoms quickly stimulating Google and Twitter activity followed soon after by calls to consumer health helplines, days off from school, and GP consultations.

Such alternative data sources also need to be combined with suitable analytic methods that can be run routinely and easily to identify potential outbreaks, so they can be further investigated and acted on if control measures are needed. This research has identified models with autoregressive information as promising approaches for the analysis of a set of alternative data sources. However, for waterborne outbreaks, as in Havelock North, inclusion of measures from drinking water supply and weather conditions could be included as further data sources for disease surveillance.

This study used the traditional ARIMA models to assess the efficiency of using alternative data sources for the early prediction of a large Campylobacter outbreak. The development of further machine learning models using other techniques to validate the results of this study will be useful. For example, deep learning–based algorithms have been found to increase the performance of traditional time series forecasting methods [46,47].

The Havelock North outbreak was very large. The signal produced in data sources was therefore easier to detect than would be the case in a smaller outbreak where the signal-to-noise ratio would be lower. It would be useful to repeat the study with outbreaks of smaller magnitude and in different settings to determine whether similar findings apply.

There are multiple operational questions that would need to be resolved before any of the methods identified here could be introduced for routine use by public health agencies in New Zealand or elsewhere. In particular, it is important to identify the range of conditions or syndromes where early detection is important for guiding effective public health action. It is also important to consider the volume of false positives that might be generated and the additional resources required to investigate and rule them out. The range of surveillance modalities also needs to be considered. For example, specific forms of environmental surveillance may be more effective for guiding public health action, for example, improved surveillance of drinking water quality and meteorological data may be more effective in preventing disease rather than focusing on early indicators of illness. Resource issues will also need to be considered, which might favor systems that are already operating on a real-time basis (eg, consumer calls to HealthLine).

### Conclusions

This study presents several important conclusions. We tested the use of data from alternative sources in predictive models and showed that they could have provided earlier detection of the Havelock North outbreak. Given the need for early intervention to curb disease transmission, our model predictions could fill a critical time gap in existing surveillance based on notification of cases of disease. These notifications inevitably do not appear until a few days after the occurrence of a communicable disease outbreak. Our results show that models that combine consumer helpline data with autoregressive information of notified case counts performed best for predictions 1 and 2 days ahead, whereas models using Google and Twitter data performed best for predictions 3 and 4 days ahead, although with lower prediction accuracy. Spatiotemporal clusters showed an earlier spike in school absenteeism and consumer helpline inquiries when compared with the notified case counts in the city primarily affected by the outbreak, which suggests that spatiotemporal modeling of alternative data sources could help to identify and locate outbreaks earlier in their development. The methods presented here can potentially be expanded to other regions in the country to signal changes in disease incidence for public health decision makers. However, before doing that, a number of key questions will need to be systematically investigated to establish the practical role of these methods and how they could be most effectively integrated into routine public health practice.

## Appendix

Multimedia Appendix 1 Symptoms classified as gastrointestinal illness in HealthLine calls.

Multimedia Appendix 2 Keywords used to collect Google Trends data.

Multimedia Appendix 3 Correlation and cross correlation of key words in Google Trends with the notified case counts.

Multimedia Appendix 4 Gnip Query to collect Twitter data.

Multimedia Appendix 5 Codes used to collect absenteeism data form primary schools.

## Abbreviations

AR: autoregressive
ARIMA: autoregressive integrated moving average
CHL: consumer helpline
GP: general practice
GT: Google Trends
RMSE: root mean square error
rRMSE: relative root mean square error
SARIMA: seasonal autoregressive integrative moving average
