# Supplementary material for: Potential Early Identification of a Large Campylobacter Outbreak Using Alternative Surveillance Data Sources: Autoregressive Modelling and Spatiotemporal Clustering
Source: JMIR Public Health Surveill. 2020 Sep 17;6(3):e18281. doi: 10.2196/18281 (PMC7530686; doi:10.2196/18281)
Supplement: Multimedia Appendix 1 [file publichealth_v6i3e18281_app1.docx]

**Multimedia Appendix 1 - Symptoms classified as gastrointestinal illness in Healthline calls**

| Abdominal Pain | Abdominal throbbing/pain | Gastroenteritis |
| --- | --- | --- |
| Abdominal cramps | Diarrhoea | Nausea |
| Abdominal swelling | Diarrhoea and vomiting | Vomiting |
