# Supplementary material for: Potential Early Identification of a Large Campylobacter Outbreak Using Alternative Surveillance Data Sources: Autoregressive Modelling and Spatiotemporal Clustering
Source: JMIR Public Health Surveill. 2020 Sep 17;6(3):e18281. doi: 10.2196/18281 (PMC7530686; doi:10.2196/18281)
Supplement: Multimedia Appendix 2 [file publichealth_v6i3e18281_app2.docx]

**Multimedia Appendix 2 - Keywords used to collect Google Trends data**

| Campylobacter | Fever | Nausea |
| --- | --- | --- |
| Chills | Gastro | Norovirus |
| Dehydration | Gastroenteritis | Puke |
| Diarrhea | Giardia | Rotavirus |
| Diarrhoea | Headache | Salmonella |
| Dizziness | Ill | Sick |
| Dizzy | Migraine | Vomit |
