# Supplementary material for: Potential Early Identification of a Large Campylobacter Outbreak Using Alternative Surveillance Data Sources: Autoregressive Modelling and Spatiotemporal Clustering
Source: JMIR Public Health Surveill. 2020 Sep 17;6(3):e18281. doi: 10.2196/18281 (PMC7530686; doi:10.2196/18281)
Supplement: Multimedia Appendix 3 [file publichealth_v6i3e18281_app3.docx]

**Multimedia Appendix 3 - Correlation and cross correlation of key words in Google Trends with the notified case counts**

| keywords | same  day | 1 day lag | 2 days lag | 3 days lag | 4 days lag | 5 days lag | 6 days lag | 7 days lag | 8 days lag | 9 days lag | 10 days lag |
| --- | --- | --- | --- | --- | --- | --- | --- | --- | --- | --- | --- |
| Campylobacter | 0.24 | 0.26 | 0.19 | 0.1 | 0.05 | 0.02 | 0.01 | 0.02 | 0.02 | 0 | -0.02 |
| diarrhea | 0.03 | 0.07 | 0.06 | 0.01 | -0.02 | -0.03 | -0.03 | -0.04 | -0.03 | -0.03 | -0.04 |
| diarrhoea | 0.15 | 0.14 | 0.05 | 0 | -0.01 | -0.01 | -0.02 | -0.02 | -0.01 | -0.01 | -0.03 |
| gastro | 0.23 | 0.26 | 0.23 | 0.27 | 0.22 | 0.2 | 0.13 | 0.05 | 0 | -0.01 | -0.01 |
| Gastroenteritis | 0.18 | 0.29 | 0.31 | 0.28 | 0.21 | 0.33 | 0.29 | 0.14 | 0.03 | 0 | 0 |
| puke | 0 | 0.02 | 0.09 | 0.16 | 0.26 | 0.28 | 0.25 | 0.19 | 0.29 | 0.26 | 0.13 |
| vomiting | -0.04 | -0.04 | -0.04 | -0.04 | -0.04 | -0.03 | -0.01 | 0.01 | 0.03 | 0.04 | 0.03 |
