# Supplementary material for: Potential Early Identification of a Large Campylobacter Outbreak Using Alternative Surveillance Data Sources: Autoregressive Modelling and Spatiotemporal Clustering
Source: JMIR Public Health Surveill. 2020 Sep 17;6(3):e18281. doi: 10.2196/18281 (PMC7530686; doi:10.2196/18281)
Supplement: Multimedia Appendix 4 [file publichealth_v6i3e18281_app4.docx]

**Multimedia Appendix 4 - Gnip Query to collect Twitter data**

(”a cold” OR “abdominal cramps” OR “abdominal pain” OR “Abdo pain”  OR “body aches” OR “body weakness” OR breathless OR campylobacter OR chills OR chucking OR “cold sweating” OR crook OR “crook stomach” OR dehydration OR contains:diarrh OR drowsy OR fever OR Giardia OR headache OR “high temperature” OR “loose stools” OR “loss of appetite” OR migraine OR nausea OR norovirus OR rotavirus OR salmonella” OR contains:shiver OR “shortness of breath” OR contains:sick OR snotty OR “sore tummy” OR “stomach bug” OR “stomach upset” OR “the runs” OR “the trots” OR “throw up” OR “tummy bug” OR “tummy pain” OR “tummy ache” OR “gut ache” OR “upchuck” OR “upset stomach” OR contains:vomit OR “watery stools” OR contains:gastro OR “spewing” OR “sore guts”~2 OR “abdominal swelling” OR “the shits” OR dizzy OR dizziness OR unwell OR ill OR “off school” OR “going at both ends” OR squirts OR squirtz) -is_retweet (lang:en) (place_country:nz AND has:geo) OR (profile_country:nz AND has:profile_region)
