# Supplementary material for: Potential Early Identification of a Large Campylobacter Outbreak Using Alternative Surveillance Data Sources: Autoregressive Modelling and Spatiotemporal Clustering
Source: JMIR Public Health Surveill. 2020 Sep 17;6(3):e18281. doi: 10.2196/18281 (PMC7530686; doi:10.2196/18281)
Supplement: Multimedia Appendix 5 [file publichealth_v6i3e18281_app5.docx]

**Multimedia Appendix 5 - Codes used to collect absenteeism data form primary schools.**

| **Code** | **Short description** |
| --- | --- |
| M | Not in class – student absent due to short-term  illness/medical reasons. |
| S | Not in class – sickbay. |
| ? | Not in class – unknown reason. |
| E | Not in class – Student is absent with and explained but unjustified reason |
| J | Not in class – Justified absence, reason for absence within the school policy |
| T | Not in class – no information provided, truant. |
